# Sustained delivery of the bone morphogenetic proteins BMP-2 and BMP-7 for cartilage repair and regeneration in osteoarthritis

**DOI:** 10.1016/j.ocarto.2022.100240

**Published:** 2022-02-08

**Authors:** Ciara Whitty, Christian Pernstich, Charlotte Marris, Andrew McCaskie, Michael Jones, Frances Henson

**Affiliations:** a Cell Guidance Systems, Maia Building, Babraham Research Campus, Cambridge, CB22 3AT, UK; b Division of Trauma & Orthopaedic Surgery, University of Cambridge, Addenbrooke's Hospital, Box 180, Hills Road, Cambridge, CB2 0QQ, UK

**Keywords:** PODS, BMP-7, BMP-2, Sustained release, Joint osteoarthritis, Cartilage regeneration, PODS, Polyhedrin delivery system, BMP, Bone morphogenic protein

## Abstract

**Objective:**

Attempts to utilise growth factors (GF) such as bone morphogenic proteins (BMPs) to treat osteoarthritis (OA) in the clinic have not secured widespread adoption. However, the novel crystalline GF formulation called PODS offers new perspectives. This study investigated the hypothesis that Polyhedrin Delivery System (PODS) BMP-2 and PODS BMP-7, compared with conventional BMP-2 and BMP-7 increase capacity for cartilage repair.

**Design:**

Sustained release from PODS BMP-2 and PODS BMP-7 and their effects on OA patient-derived cells as well as a chondrocyte cell line were first assessed *in vitro.* Here, extra cellular matrix (ECM) protein gene expression and actual ECM deposition were measured and compared to the effect achieved with conventional, soluble BMPs. Subsequently, in an established murine model of cartilage regeneration of the knee joint, changes were traced over 8 weeks and scored with two metrics, modified Pineda and Mankin.

**Results:**

Both crystalline PODS BMP formulations strongly induced proliferation in primary as well as immortal cell line chondrocytes, outperforming conventional soluble BMP-2 and BMP-7. Furthermore, ECM-producing genes were upregulated and the production of ECM could be demonstrated. In the murine cartilage regeneration model, both PODS BMP-2 and PODS-BMP-7 improved cartilage repair assessed with both histological scoring methods.

**Conclusions:**

This study showed that the sustained release of GF from PODS BMPs is effective in promoting chondrogenesis *in vitro*. The small animal data suggests that this novel approach of delivering therapeutic proteins sustainably and locally to the knee has promise for developing future disease-modifying therapies of OA.

## 1 Introduction

Osteoarthritis (OA), is a progressive and degenerative joint disease, characterised by cartilage degradation, synovial inflammation, and changes to subchondral bone [1]. Globally, an estimated 300 million people suffer from hip and knee OA in 2017 [2], and in Organization for Economic Cooperation and Development (OECD) countries, 500,000 hip and joint replacement operations are performed each year. 70% of patients with OA report persistent pain despite taking prescribed medication, with 12% describing their pain as often unbearable, impacting their quality of life and work capacity [3].

Currently there are no disease-modifying or preventative treatments for OA. Consequently, the focus is on managing the primary symptoms through lifestyle changes, pain relief, anti-inflammatory medications, and physiotherapy [4]. If management is ineffective, joint replacement surgery is the conventional intervention. Joint replacement has a higher lifetime risk of revision if the primary surgery is performed at ages 50–55 where it is 33%, compared to 1–5% in the over 70s [5]. There is a clear and unmet need to treat cartilage damage at an earlier stage.

Bone morphogenetic proteins (BMPs), such as BMP-2 and BMP-7, have been implicated in cartilage homeostasis and repair, and are promising OA disease-modifying candidates. Several studies have demonstrated that these BMPs stimulate an anabolic response in cartilage explants and articular chondrocytes, promote recruitment of chondroprogenitors, and up-regulate synthesis of extracellular matrix (ECM) components including collagen and proteoglycan [[6], [7], [8]]. Furthermore, BMP-2 and BMP-7 have been shown to be chondroprotective in small and large animal models of OA [[9], [10], [11], [12], [13], [14], [15]].

However, the use of conventional recombinant BMP-2 (rBMP-2) and rBMP-7 as therapeutic agents has been limited by their instability, with reported half-lives ranging between minutes and hours [16,17]. Furthermore, the utility of incorporation into scaffolds such as PLGA is limited by protein denaturation and burst release [18,19]. Consequently, high doses are required to achieve efficacy, which can lead to toxicity and off-target side effects [16].

The Polyhedrin Delivery System (PODS®) is a sustained protein release technology which has been developed to overcome these limitations. PODS® technology harnesses polyhedrin protein that produces a complex, highly organised crystal scaffold which encases cypovirus progeny virions [20,21]. This mechanism has been adapted to generate PODS® proteins, (micron scale co-crystals synthesised within insect cells by co-expression of polyhedrin and a cargo protein incorporated via an immobilization tag which binds the polyhedrin protein) [[22], [23], [24]]. Constraint of a growth factor within the crystal allows for long-term stable capture of functional, biologically active growth factor. Degradation of PODS® crystals, mediated by cell- and protease-dependent mechanisms, enables release of cargo protein at physiologically-relevant levels over a period of weeks-to-months [[25], [26], [27]].

Here, we report a proof-of-concept study to assess whether PODS® BMP-2 (pBMP-2) and PODS® BMP-7 (pBMP-7) promote cartilage repair to treat OA. The proliferation of chondrocytes and production of cartilage ECM *in vitro* were first investigated to confirm efficacy of PODS® compared with conventional recombinant growth factor, and to provide guidance on dosage *in vivo*. Administration of PODS® by intra-articular injection into the knee joint was performed to assess whether pBMP-2 and pBMP-7 heal a chondral defect in an *in vivo* murine model of cartilage repair.

## 2 Materials and methods

### 2.1 Synthesis of pBMP-2 and pBMP-7

pBMP-2 and pBMP-7 were synthesised as described [25,26] using full-length BMP-2 and BMP-7 protein (NCBI accession numbers P12643 and P18075) fused to the H1 incorporation tag [28].

Transfer DNA was co-transfected into *Spodoptera frugiperda 9* (Sf9) cells with linearised baculovirus (BV) DNA using TransIT®-Insect (Mirus Bio, Madison, WI). Replication-competent BV was rescued by recombination between the transfer vector and linearised viral DNA. Virus was harvested and plaque purification performed to isolate a single recombinant BV. Plaques were screened and BV was harvested to infect Sf9 cells to produce PODS® crystals. Subsequently, crystals were harvested and purified by lysing Sf9 cells using successive rounds of sonication and PBS washes. Purified PODS® were sterility tested and lyophilised.

### 2.2 Primary chondrocyte isolation

Primary human articular chondrocytes were isolated from cartilage obtained from donors undergoing total knee arthroplasty for osteoarthritis. Tissue was collected under ethical consent obtained from the Local Ethics committee and the UK Home Office (Project Licence number 70/8635). Articular cartilage was shaved from the subchondral bone, and washed in PBS. Chondrocytes were isolated by further mincing the cartilage and incubating with digestion buffer at 37 ​°C, 5% O_2_ on a shaking platform (55 ​rpm). Digestion buffer consisted of Dulbecco's Modified Eagle medium (DMEM) (1 ​g/l glucose) with GlutaMAX™, pyruvate and Phenol Red (ThermoFisher Scientific, Waltham MA) supplemented with 10% foetal bovine serum (FBS) (First Link, Wolverhampton, UK), 100 units/ml penicillin and 100 μg/ml streptomycin (P/S) final concentration (ThermoFisher Scientific), and 6 ​mg/ml collagenase A (Sigma Aldrich). After digestion, the cell suspension was filtered through a 70 ​μm MACS SmartStrainer (Miltenyi Biotec, Bergisch Gladbach, Germany), centrifuged at 300*×g* and washed with PBS.

### 2.3 Cell culture

Isolated OA primary chondrocytes were expanded in DMEM (1 ​g/l glucose) with GlutaMAX™, pyruvate and phenol red supplemented with 10% FBS and 1xP/S. Chondrocytes were cultured at 37 ​°C up to passage 3 in hypoxia (3% O_2_) using a HeraCell™ Vios Tri-Gas Incubator (Fisher Scientific, Pittsburgh, PA).

Clonetics™ Normal Human Articular Chondrocytes (NHAC-kn) isolated from a six-year old female (lot number 6F4018, Lonza, Basel, Switzerland) were expanded in monola*y*er in DMEM (1 ​g/l glucose) with GlutaMAX™, pyruvate and phenol red (ThermoFisher Scientific) supplemented with 10% foetal bovine serum (FBS) (First Link UK) and 1x penicillin-streptomycin-glutamine (PSG). Following the manufacturer's guidelines, NHAC-kn cells were cultured to passage 15.

To promote extracellular matrix (ECM) production, primary OA chondrocytes and NHACs were seeded into wells of flat-bottomed hydrophilic 24-well plates (Sarstedt) as a micromass of 2 ​× ​10^5^ ​cells (10 μl/well). After incubating for 2 ​h at 37 ​°C, primary OA chondrocytes in hypoxic and NHACs in normoxic conditions, 250 ​μl of media was slowly added. Cells were treated with conventional rBMP-2 (100 ​ng/ml), conventional rBMP-7 (200 ​ng/ml), PODS® Empty (pEmpty) (50 ​ng/ml), pBMP-2 (25–200 ​ng/ml), or pBMP-7 (25–200 ​ng/ml) (all Cell Guidance Systems, Cambridge UK). A half media change was performed every 3–4 days for PODS®-containing wells (with no addition or replacement of crystals), or a half media change with replacement of growth factor for wells containing conventional rBMP-2 or rBMP-7.

### 2.4 qRT-PCR

Total cellular RNA was isolated from cells after 7 days of micromass culture using the RNeasy® Mini Kit (Qiagen), following the manufacturer's protocol, including an on-column gDNA elimination treatment (Qiagen). RNA concentration and quality was quantified using UV-spectrometry with the Nanodrop ND-2000 Spectrophotometer and stored at −80 ​°C. Reverse transcription was performed using the QuantiTect Reverse Transcription Kit (Qiagen). qRT-PCR was performed using 2x Fast SYBR Green (ThermoFisher Scientific) with qPCR primers (ThemoFisher Scientific)_for the following human genes: collagen type1 (COL1A1) GCTGCTGTCTTTGTCACCCACA and CAGGAAACAGCTATGACC, aggrecan (ACAN) TGTAAAACGACGGCCAGT and CAGGAAACAGCTATGACC, and hypoxanthine-guanine phosphoribosyltransferase (HPRT) TGTAAAACGACGGCCAGT and CAGGAAACAGCTATGACC and β-actin (BACT) TGTAAAACGACGGCCAGT and CAGGAAACAGCTATGACC (housekeeping genes)). The collagen type II (COL2) forward and reverse primers sequences were 5′-TGGGTGTTCTATTTATTTATTGTCTTCCT-3′ and 5′-GCGTTGGACTCACACCAGTTAGT-3′, respectively (Integrated DNA Technologies, Coralville, Iowa). PCR conditions were 95 ​°C for 20 ​s, followed by 40 cycles of 95 ​°C for 3 ​s and 60 ​°C for 30 ​s.

### 2.5 Alcian blue

Alcian blue staining was assessed after 21 days of micromass culture. Cells were washed with PBS and fixed in 10% formalin for 20 ​min. After fixing, cells were washed with PBS before adding 1% Alcian blue (Sigma, St Louis, Missouri) in 0.1 ​M HCl. After overnight, room-temperature incubation, cells were washed three-times with 0.1 ​M HCl and washed with distilled water.

### 2.6 Real-time cell proliferation

Real-time monitoring of cell proliferation was performed using the xCELLigence® RTCA DP instrument (Agilent Technologies). Primary OA chondrocytes or NHAC-kn cells were serum starved overnight in DMEM +1% FBS. On the next day, xCELLigence E-plate wells (Agilent Technologies) were filled with 50 ​μl of DMEM ​+ ​1% FBS and incubated at room temperature for 2 ​h. The serum-starved cells were seeded at 10^3^ ​cells per well and further incubated at room temperature for 30 ​min. Finally, cultures were treated with either conventional rBMP-2 (100 ​ng/ml); conventional rBMP-7 (200 ​ng/ml); pEmpty (200 ​ng/ml equivalent); pBMP-2 (25–200 ​ng/ml); or pBMP-7 (25–200 ​ng/ml). Proliferation was monitored for up to 14 days without any media changes or further supplements.

### 2.7 *In vivo* murine model of cartilage regeneration

A chondral lesion in the patella groove was generated as previously described by Eltawil et al., 2009 [29] in eight-week-old female C57/BL6 mice. For each animal the right knee joint of one leg was operated on under general anaesthesia. The animal was placed in dorsal recumbency and a medial approach to the stifle was made by sharp incision. Following dislocation of the patella a full thickness chondral defect was produced with a 26 ​g needle. The patella was replaced and the subcutaneous tissues and skin sutured in separate layers. PODS® (with/without growth factors and in the presence/absence of a carrier hydrogel), were injected intra-articulalry to assess the effect on the healing of the chondral defect after Week 4 and Week 8.

The joint was injected with 1 ​μl of each treatment (n ​= ​5 mice per group) shown in Table 1 - Murine model treatment groups, using a 2 ​μl Hamilton syringe (Sigma Aldrich). 6.75 ​× ​10 [5] PODS® crystals (equivalent to 18 ​ng of conventional recombinant protein) were administered for each treatment group either in PBS or in 0.5% (v/v) peptide-based hydrogel (PuraStat® [3D Matrix Medical Technology], Tokyo, Japan), with pEmpty and 0.5% hydrogel alone serving as negative controls. After creation of the defect, the patella was repositioned and sutured (Ethicon, Raritan, New Jersey). The contra-lateral knee was non-operated.

**Table 1:** Murine model treatment groups. Treatment groups for each PODS (p) condition used.

	pBMP-2 ​+ ​hydrogel	pBMP-7 ​+ ​hydrogel	pEmpty ​+ ​hydrogel	pBMP-2 (PBS)	pBMP-7 (PBS)	pEmpty (PBS)	Hydrogel
4 ​Weeks	5	5	5	5	5	5	5
8 ​Weeks	5	5	5	–	–	–	–

### 2.8 Histology

Histological analysis was performed at Week 4 to assess the response of the joint to the administration of intra-articular PODS® and at Week 8 post-surgery to assess the rate of joint healing and OA using industry-conventional scoring methods. Animals were humanely sacrificed at Week-4 and Week-8 post-surgery and stifles retrieved. Knee joints were fixed and decalcified in 10% EDTA pH 8 for 14 days. The joints were processed sequentially through ethanol and xylene immersions using a Leica TP1020 Semi-enclosed Benchtop Tissue Processor and paraffin embedded using the HistoCore Arcadia (Leica Biosystems, Wetzlar, Germany). Joints were serially sectioned at 7 ​μm intervals using the Leica Biosystems RM2245 Semi-Automated Rotary Microtome. Three sections per animal were scored from the middle of the defect.

Sections were stained with Safranin O. After deparaffinisation and rehydration, sections were stained with Weigert's iron haematoxylin (Sigma Aldrich) working solution for 10 ​min, followed by washing with water for 10 ​min. Slides were transferred to 0.1% Fast Green FCF (Sigma Aldrich) for 5 ​min, before being transferred to 1% acetic acid for 10–15 ​s. Subsequently, slides were stained in 0.1% Safranin O solution for 5 ​min. Sections were then dehydrated through 100% ethanol, cleared with xylene and mounted using DPX mounting medium. Slides were analysed with a Nikon Eclipse Ti inverted Microscope, and images captured with an Orca OSG camera (Nikon, Japan) using NIS-Elements Advanced Research software.

Joint surface repair of the chondral defect was scored using the Modified Pineda scoring system [30] and OA in the joint was scored using the Mankin scoring system [31,32]. The entire joint was sectioned and three slides per animal were scored from the middle of the lesion. Scoring was performed independently by two observers blinded to the group assignment.

### 2.9 Statistical analysis

A *t*-test was used to identify statistical differences between groups.

## 3 Results

### 3.1 PODS® BMP-2 and PODS® BMP-7 stimulate chondrocyte proliferation

**Fig. 1:**
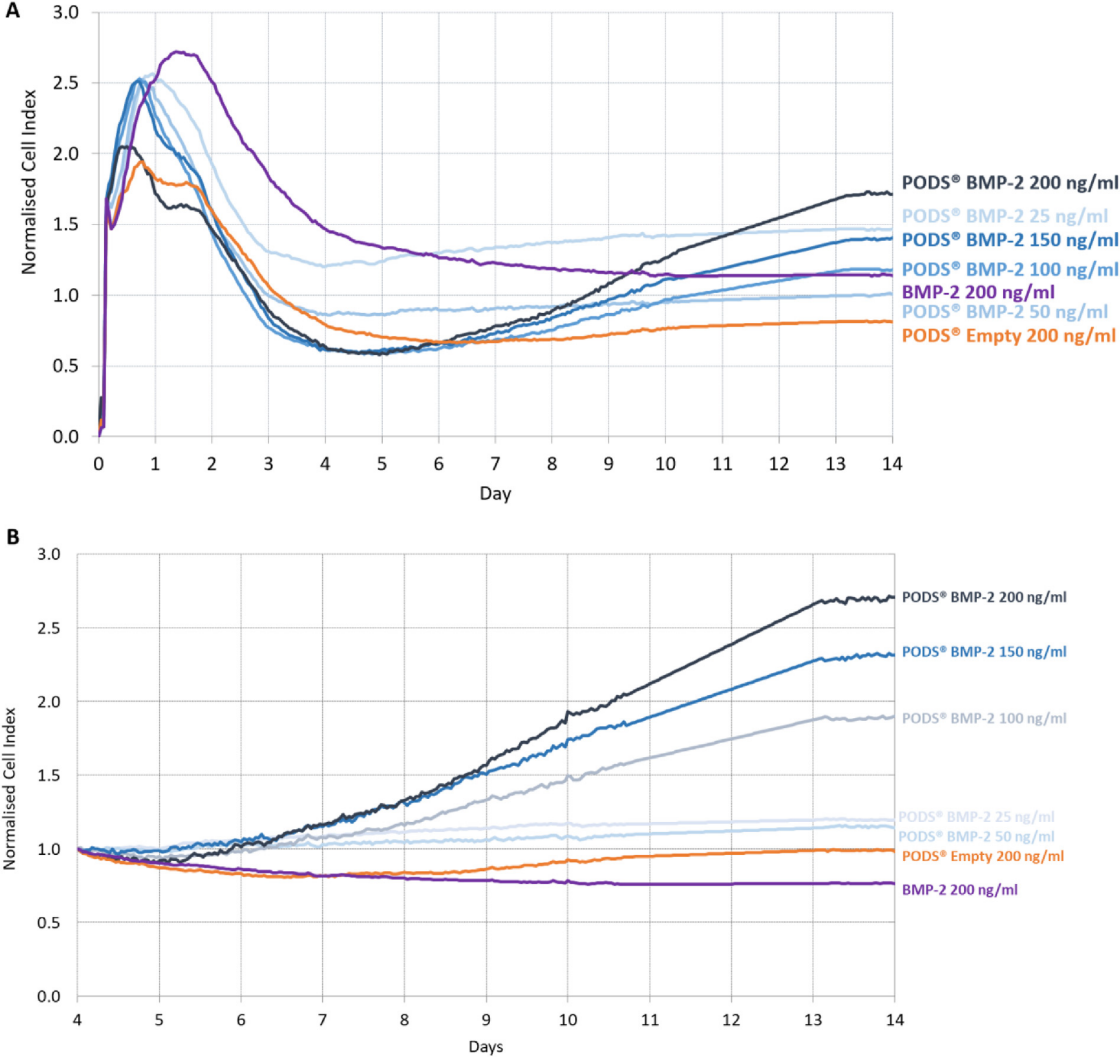
Effect of PODS® Bone morphogenic protein-2 (BMP-2) on real-time chondrocyte proliferation. Cell proliferation was monitored in real-time using xCELLigence's E-system. This passes an electrical current through a plate and measures impedance. As cells grow, the impedance is modulated by the amount of contact with the plate. This value is converted to a CI, a proxy for cell number. index. Chondrocytes were cultured up to 14 days with PODS-BMP-2 (pBMP-2) (25–200 ​ng/ml equivalent), alongside pEmpty (200 ​ng/ml equivalent), and recombinant BMP-2 (200 ​ng/ml). No media changes were performed over the entire culture period. CI is normalised to the initial cell seeding peak at 4 ​h in (A) and Day 4 in (B). Traces represent the mean CI for each treatment group.

**Fig. 2:**
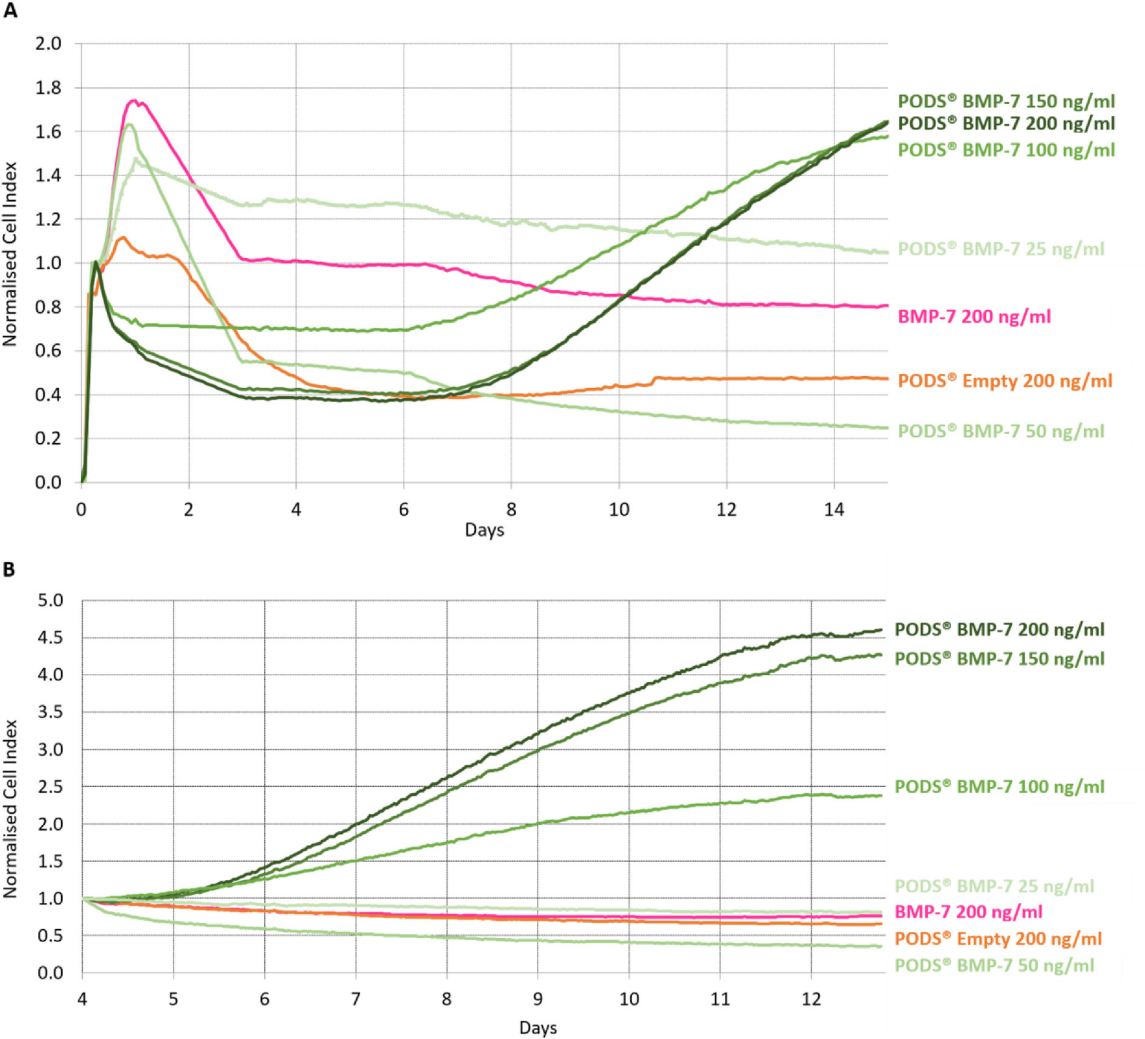
Effect of PODS® Bone morphogenic protein-7 (BMP-7) on real-time chondrocyte proliferation. Cell proliferation was monitored in real-time using xCELLigence's E-system. This passes an electrical current through a plate and measures impedance. As cells grow, the impedance is modulated by the amount of contact with the plate. This value is converted to a CI, a proxy for cell number. index. Chondrocytes were cultured up to 14 days with PODS-BMP-7 (pBMP-7) (25–200 ​ng/ml equivalent), alongside pEmpty (200 ​ng/ml equivalent), and recombinant BMP-7 (200 ​ng/ml). No media changes were performed over the entire culture period. CI is normalised to the initial cell seeding peak at 4 ​h in (A) and Day 4 in (B). Traces represent the mean CI for each treatment group.

The xCELLigence assay monitored real-time changes in cellular proliferation and viability in the presence of pBMP-2 (Fig. 1) or pBMP-7 (Fig. 2) compared to their conventional recombinant counterparts. Cells were seeded into wells of plates incorporating surface microelectrodes. The instrument measures across each well the impedance, governed by the total cell surface area in contact with the electrodes, which is representative of the total cell number, viability and morphology and calculated as the arbitrary unit Cell Index (CI) by the instrument's software. Chondrocytes (either primary or NHAC-kn) were cultured with between 9 ​× ​10 [5]–7.2 ​× ​10 [6] pBMP-2 or pBMP-7 (equivalent to 25–200 ​ng/ml conventional recombinant growth factor), to assess any effect of PODS® on proliferation. In comparison to conventional rBMP-2 (100 ​ng/ml), rBMP-7 (200 ​ng/ml), and pEmpty (25 or 200 ​ng/ml equivalent). Chondrocytes were cultured up to 14 days with no media changes. CI was normalised to the initial cell seeding peak at 4 ​h (Fig. 1, Fig. 2A) or Day 4 (Fig. 1, Fig. 2B).

Over the first two days, conventional rBMP-2 or rBMP-7 generated a sharp increase in proliferation, with an increase in normalised CI up to 2.7 and 1.7, respectively. Chondrocyte proliferation was similar for both pBMP-2 and pBMP-7. For these groups, as well as for pEmpty, there was a slight increase in normalised CI over this period, with a higher peak for the lower numbers of PODS®, possibly related to initial post-seeding changes in cell impedance. After the first two days of culture, there was a decline in normalised CI that reached a minimum in all treatment groups at around day 4. This indicates that the slow-reacting chondrocytes have gradually settled, spread and undergone morphological changes, commonly observed in RTCAs. Normalizing to the CI minimum at Day 4 allowed for a more thorough assessment of how cells reacted to each treatment for the remaining 10 days of the assay.

Between Day 4 and Day 14, with higher concentrations of pBMP-2 and pBMP-7 (100, 150 and 200 ​ng/ml equivalent) there was a steady increase in normalised CI to Day 14, with a peak average normalised CI of 1.8–2.7 for pBMP-2, and 2.2–4.6 for pBMP-7. This augmentation in normalised CI was more marked for the two highest doses of PODS®. By contrast, there was no increase in normalised CI between Days 4 and 14 for rBMP-2, rBMP-7, pEmpty, and the lower concentrations of pBMP-2 or pBMP-7 (25 ​ng/ml and 50 ​ng/ml).

Chondrocytes can act as non-professional phagocytes [33,34]. We observed chondrocytes phagocytosing PODS® proteins efficiently, regardless of their cargo protein (supplementary Figure S1).

### 3.2 *PODS*® *BMP-2 and PODS*® *BMP-7 induce ECM genes and proteoglycan synthesis*

**Fig. 3:**
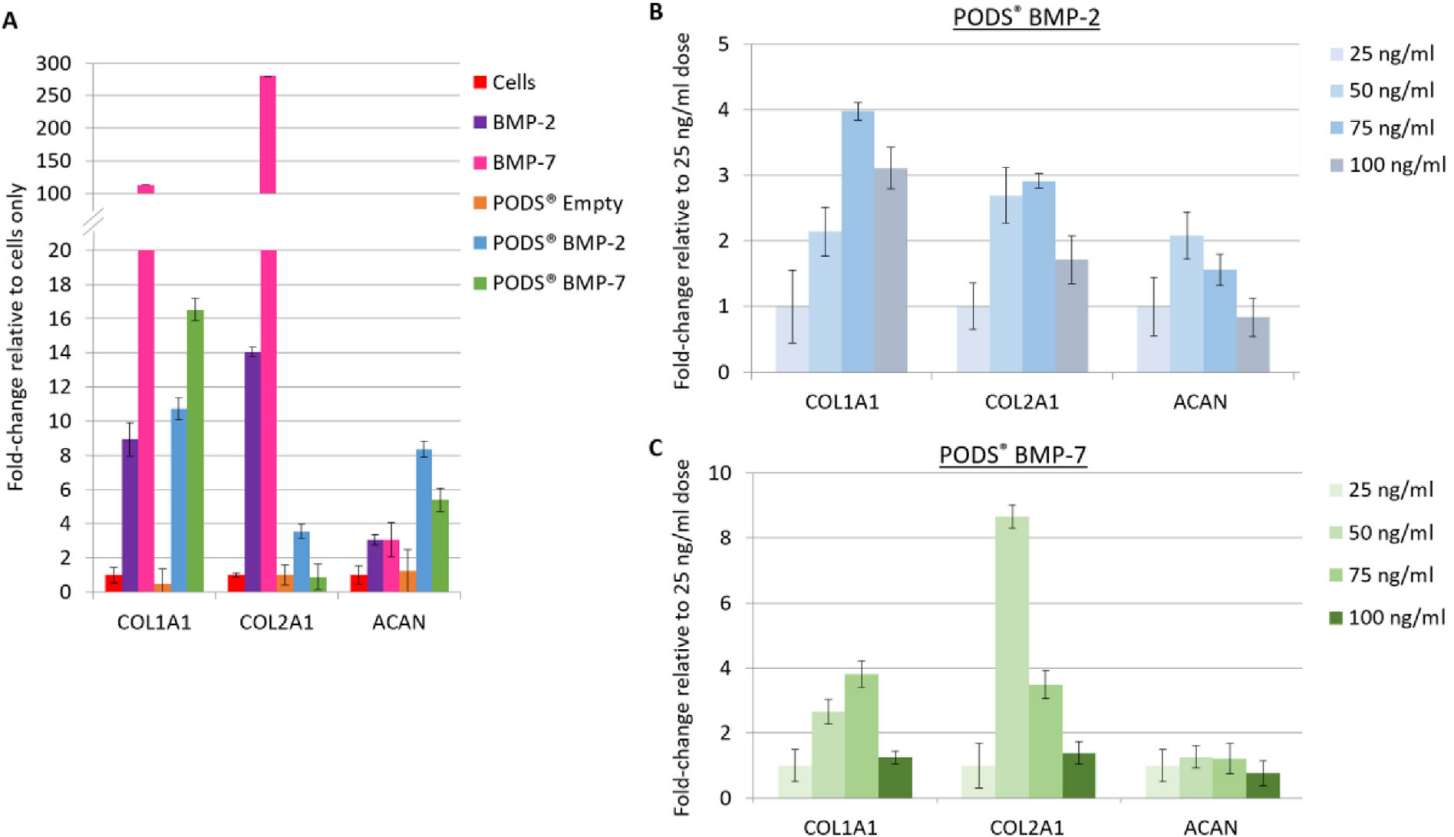
Influence of PODS® growth factors on extracellular matrix (ECM) mRNA expression. qRT-PCR was performed to measure ECM marker expression in micromass chondrocytes cultured over 7 days. Fig. 3A shows the relative expression when cells were cultured with pBMP-2 or pBMP-7 (both 50 ​ng/ml equivalent), alongside pEmpty (50 ​ng/ml equivalent), conventional rBMP-2 (100 ​ng/ml) or conventional rBMP-7 (200 ​ng/ml). Fig. 3B and C illustrates the relative expression of ECM markers when chondrocytes were cultured with a dose range of pBMP-2 or pBMP-7 (25–100 ​ng/ml equivalent), respectively. PCR cycle threshold (CT) values for each gene and each treatment were first individually normalised to the CT values of the housekeeping gene BACT, then normalised to either cells only (Fig. 3A) or 25 ​ng/ml dose treatment (Fig. 3B and C). A half media change was performed on Day 3 of the culture period, where conventional recombinant growth factor was replenished while no additional PODS® crystals were added to PODS®-treated wells. Points and error bars represent the mean and conventional deviation.

**Fig. 4:**
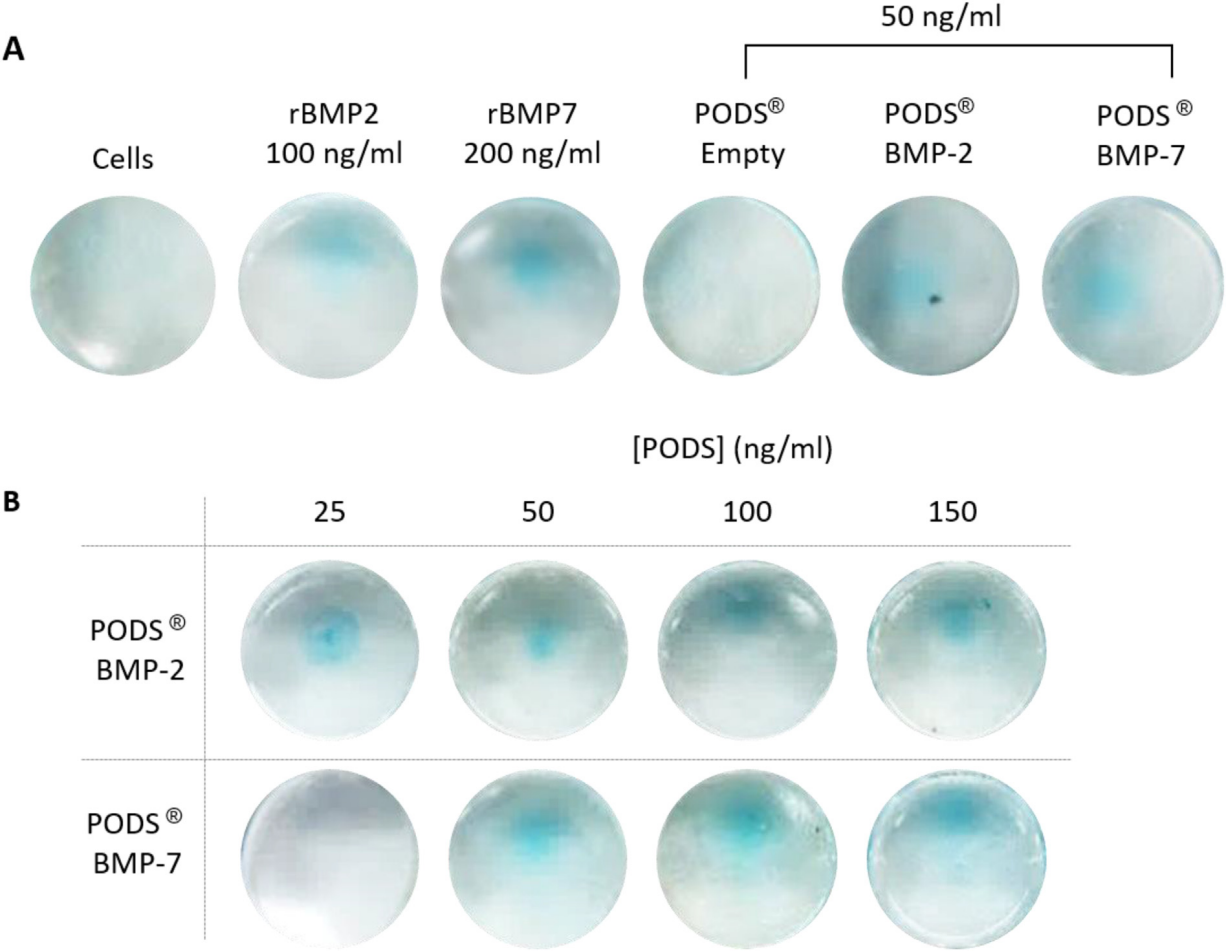
Alcian blue staining of chondrocytes. Alcian blue staining was performed to measure glycosaminoglycans (GAG) content after micromass chondrocytes were cultured over 21 days. Fig. 4A shows representative staining after culturing with pBMP-2 or pBMP-7 (both 50 ​ng/ml equivalent), alongside pEmpty (50 ​ng/ml equivalent), conventional rBMP-2 (100 ​ng/ml) or conventional rBMP-7 (200 ​ng/ml). Fig. 4B shows representative staining after culturing with a dose range of pBMP-2 or pBMP-7 (25–150 ​ng/ml equivalent) (Fig. 4B). A half media change was performed every 3–4 days during the culture period, where conventional recombinant growth factor was replenished while no additional crystals were added to PODS®-treated wells. . (For interpretation of the references to color in this figure legend, the reader is referred to the Web version of this article.)

Chondrocytes were cultured with pBMP-2, pBMP-7, pEmpty (all 50 ​ng/ml equivalent), conventional rBMP-2 (100 ​ng/ml), or conventional rBMP-7 (200 ​ng/ml). Production of collagen type I (COL1A1), collagen type II (COL2A1) and aggrecan (ACAN) was assessed after 7 days of micromass culture by qRT-PCR (Fig. 3A). Glycosaminoglycan (GAG) production was assessed after 21 days of micromass culture using Alcian blue staining (Fig. 4A).

Alcian blue staining after 21 days of micromass culture revealed increased GAG production with all BMP treatments compared with growth media only or with pEmpty (Fig. 4). No marked differences in staining were detected between the different BMP treatments.

Generally, COL1A1, COL2A1 and ACAN mRNA expression was up-regulated in chondrocytes cultured with either conventional or PODS® BMP-2 and BMP-7, when normalizing to cells cultured in growth media only. COL1A1 mRNA expression was up-regulated by a similar amount with conventional and pBMP-2 (9-fold and 10-fold, respectively). However, COL1A1 was more strongly up-regulated by conventional rBMP-7 compared with pBMP-7 (110-fold and 17-fold, respectively). Conversely, up-regulation of ACAN mRNA was greater with pBMP-2 or pBMP-7 (8-fold and 5-fold, respectively), compared with conventional rBMP2 and rBMP-7 (both 3-fold). Lastly, there was a 14-fold and 280-fold up-regulation in COL2A1 mRNA expression with conventional rBMP-2 and rBMP-7, respectively, compared with a 4-fold up-regulation with pBMP-2 and no changes observed with pBMP-7. There was little change in COL1A1, COL2A1 or ACAN mRNA expression with pEmpty relative cells cultured in growth media only.

Changes in mRNA and Alcian blue staining were also measured in chondrocytes cultured with a dose range of pBMP-2 or pBMP-7 (Figs. 3B and 4B). Alcian blue staining was observed across a range of PODS® crystals (25–100 ​ng/ml equivalent), with no trend observed between staining intensity and amounts of crystals. There was a trend towards a dose-dependent increase in mRNA expression relative to 25 ​ng/ml equivalent of PODS® treatment. For COL1A1, there was approximately a 2-fold and 4-fold increase with 50 ​ng/ml and 75 ​ng/ml of pBMP-2 and pBMP-7, respectively. With 100 ​ng/ml, there was a 3-fold increase with pBMP-2, and no change with pBMP-7.

ACAN mRNA expression increased by 1.5–2-fold with 50 ​ng/ml and 75 ​ng/ml of pBMP-2, relative to 25 ​ng/ml pBMP-2, with no change for 100 ​ng/ml of pBMP-2. There was increase in ACAN mRNA expression with any dose of pBMP-7. Lastly, COL2A1 mRNA increased between 1.5 and 3-fold, with 50, 75 and 100 ​ng/ml pBMP-2 treatment, and 8.5-fold and 3.5-fold with 50 or 75 ​ng/ml of pBMP-7, respectively.

### 3.3 *PODS*® *BMP-2* and *PODS*® *BMP-7 promote in vivo chondral defect healing*

**Fig. 5:**
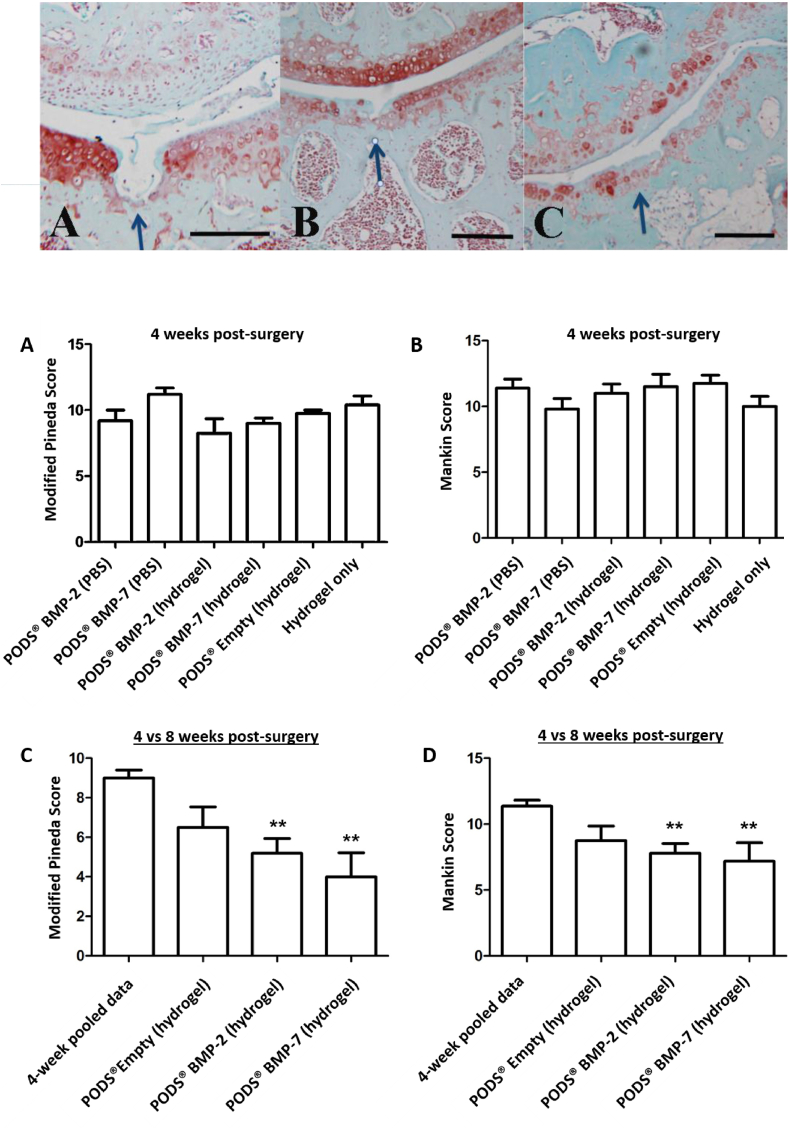
Repair of cartilage defect in murine model after intra-articular injection of PODS® crystals. Safranin-O staining of sections from injured joints of 8-week old C57BL/6 mice. A longitudinal, full-thickness cartilage defect was created in the mice with a 27G needle, after which mice were intra-articularly injected with the treatment (all n ​= ​5 per group). Mice sacrificed at eight weeks post-surgery were treated with 18.75 ​ng equivalent PODS-bone morphogenic protein-2 (pBMP-2), pBMP-7 or pEmpty in 0.5% hydrogel. Images show representative repair of cartilage for A 8w control, B 8w pBMP-2 and C 8w pBMP-7. Scale bar 100 ​μm.

**Fig. 6:**
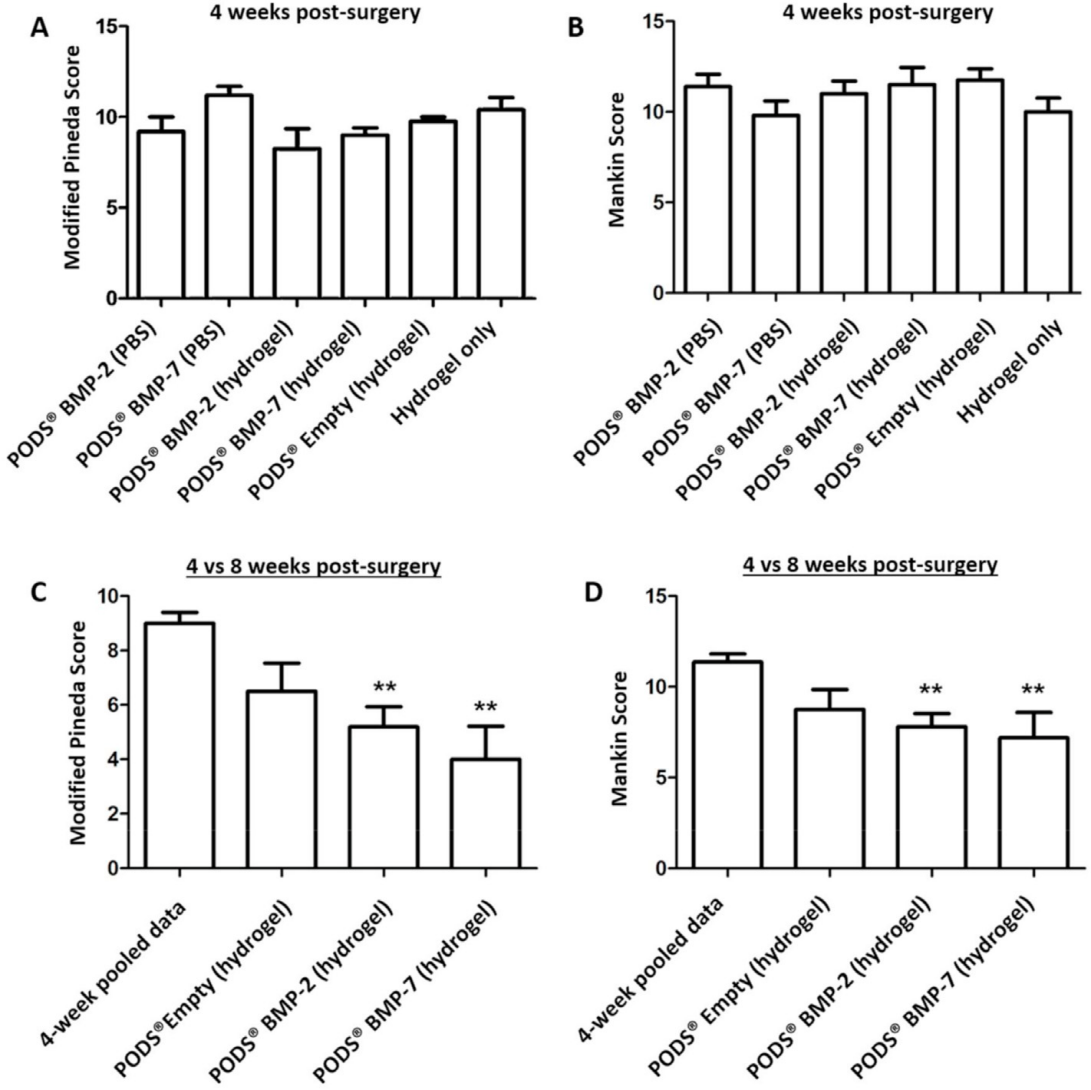
Modified Pineda and Mankin histology scoring of cartilage repair and OA in murine model after intra-articular injection with PODS® crystals. Safranin-O staining of histological sections from injured joints of 8-week old C57BL/6 mice were assessed for cartilage repair and OA using the Modified Pineda and Mankin scoring systems. A longitudinal, full-thickness cartilage defect was created in the mice with a 27G needle, after which mice were intra-articularly injected with the treatment (all n ​= ​5 per group). Mice sacrificed at Week 4 post-surgery were treated with PODS-bone morphogenic protein-2 (pBMP-2), pBMP-7, or pEmpty in PBS or 0.5% hydrogel, or 0.5% hydrogel alone. Mice sacrificed at eight weeks post-surgery were treated with pBMP-2, pBMP-7 or pEmpty in 0.5% hydrogel. 6.75 ​× ​10 [5] PODS crystals were administered per treatment group, equivalent to 18.75 ​ng of protein. Joints were fixed, sectioned and stained with Safranin O for histological analysis of cartilage repair at 4 and 8 weeks. Sections were scored by two observers blinded to the group assignment to assess the level of cartilage repair and osteoarthritis. Bars show the mean score with error bars representing the conventional error. ∗∗ indicates significant (p ​< ​0.05) improvement in histological score compared with Week 4 pooled data.

Mice were sacrificed at Week 4 and Week 8 post-surgery, and sectioned joints stained with Safranin O (Fig. 5). Sections were quantitatively assessed for evidence of inflammation and the extent of cartilage repair and osteoarthritis in the joints using the Modified Pineda score and Mankin score, respectively. At week 4 post-surgery there was no significant difference in the damage repair between the different groups with either scoring system (Fig. 6A and B), with the mean joint score between 8.25 and 11.2 (Pineda score) and 9.8 and 12 (Mankin Score). We did not observe any adverse inflammatory reaction in the joint.

For mice that received PODS® proteins embedded in hydrogel, including the control pEmpty, the study was continued to week 8 post-surgery. For statistical reasons, scores for the negative controls (pEmpty in hydrogel and hydrogel only) were pooled. At eight weeks, there was a statistically significant reduction in the Modified Pineda score and the Mankin score in mice receiving pBMP-2 in 0.5% hydrogel or pBMP-7 in 0.5% hydrogel compared with the pooled Week 4 data, with mean scores ranging from 4.0 to 9.0 (Pineda score) and 7.2 to 11.36 (Mankin score) (Fig. 6C and D). The chondral injury model produces degeneration in the joint in the strain of mouse used in this experiment and both the Modified Pineda and Mankin score will worsen over time if left untreated (Eltawil et al., 2009). That the scores reduced in the presence of pBMP-2 and 7, despite the later time point, is clear evidence of the therapeutic effect of the treatment.

## 4 Discussion

With important roles in cartilage homeostasis and repair, BMP-2 and BMP-7 are promising disease-modifying candidates to treat OA. This proof-of-concept study demonstrates the efficacy and safety of pBMP-2 and pBMP-7 *in vitro* as well as *in vivo* to promote cartilage repair. This work builds on previous data where pBMP-2 incorporated into an absorbable collagen sponge (ACS) promoted bone formation to repair a critical-sized defect in a rat calvaria model, with superior efficacy to conventional rBMP-2 [26]. Furthermore, incorporation of pBMP-2 into an atelocollagen sponge facilitated the regeneration of new bone in the mandibular alveolar bone ridge [35].

Culturing primary OA chondrocytes with pBMP-2 or pBMP-7 up-regulated ECM marker genes (collagen 2 and aggrecan) and synthesis of GAGs, which play crucial roles in the function of articular cartilage. Unexpectedly, expression of COL1A1, a marker of de-differentiation into a fibroblast-like phenotype and usually found in subchondral bone tissue, was also increased in all treatments except pEmpty. An elevated non-chondrogenic COL1A1 expression is generally accompanied by a down-regulation of COL2A2. And although not uncommon in low-quality chondrocyte *in vitro* cultures, high COL1A1 is also indicative of OA tissue such as isolated OA chondrocytes used in this study.

Chondrogenic differentiation through Alcian blue staining with pBMP-2 supports previously published data [26]. Whilst Alcian blue staining did not reveal any differences between PODS® growth factor and conventional recombinant growth factor, there were some differences in mRNA expression. For example, expression of ACAN was higher with both pBMP-2 and pBMP-7 compared with conventional recombinant growth factor, whereas expression of COL2A1 was higher for conventional rBMP-2 and rBMP-7 compared with pBMP-2 and pBMP-7. The half media change for the PODS®-containing wells would have removed half of the released growth factor from the culture system, which would take time to replace compared to the instant replenishment for conventional recombinant growth factor-containing wells.

The effect of not changing the media during the culture period on PODS®-containing wells is illustrated by the real-time proliferation results. BMP-2 and BMP-7, respectively, were continuously released from PODS® crystals enabling sustained cellular proliferation over a period of two weeks, including an initial lag phase of four days. We also observed that PODS® crystals are phagocytosed by chondrocytes over approximately 72 ​h. The degradation of PODS crystals intracellularly may release BMPs to act either internally or externally, after secretion. It remains unclear which mode of action is responsible for the observed BMP-specific stimulation of proliferation.

The proliferative activity of PODS® was in contrast to conventional recombinant BMP-2 and BMP-7 counterparts, which only promoted chondrocyte proliferation for the first two days of culture. This emphasizes the advantages of PODS®, which constantly release functional growth factor at physiologically-relevant levels over a sustained period of time. These *in vitro* results support findings from previous studies which have shown that cells cultured with rBMP-2 or rBMP-7 stimulate an anabolic response through synthesis of extracellular matrix (ECM) components including collagen and proteoglycans, induce cell proliferation, and promote chondrogenic differentiation [[6], [7], [8],[36], [37], [38], [39], [40], [41]].

In the second part of the study, pBMP-2 and pBMP-7 crystals, respectively, were injected into the intra-articular knee joint space of 5 mice for each group of a conventional OA mouse model study. The effects of a single dose of PODS were observed for up to 8 weeks and changes to the cartilage assessed using the Modified Pineda and Mankin scoring systems. Histological analysis of damage regeneration after 4 weeks did not show a difference between the study arms. This was not entirely unexpected, as cartilage regeneration is a multi-stage process involving chondrocyte proliferation and ECM production, both of which are generally slow. Analysis of the joints 8 weeks after administering treatment demonstrated that both pBMP-2 and pBMP-7 improved healing of the chondral defect and reduced signs of osteoarthritis, such as articular cartilage integrity as judged by the Mankin score. Moreover, no adverse side effects were observed, in either PBS or 0.5% hydrogel formulations. Our results are in agreement with data from other *in vivo* studies which have investigated the effects of rBMP-2 and rBMP-7 for treatment of OA or cartilage repair in small and large animal models including dog, rabbit, sheep, goat and pony, although in our study the dose required was considerably smaller [[9], [10], [11], [12], [13], [14], [15],[42], [43], [44], [45], [46], [47]].

Conventional rBMP-2 and rBMP-7 are unstable, with sera half-lives ranging between minutes and hours [16,17]. Moreover, there is a lack of conventionalisation with a wide range of doses used in animal models [46]. Furthermore, the utility of incorporation into scaffolds such as PLGA is limited by protein denaturation and burst release [18,19]. As a consequence, high doses are required to achieve efficacy, which is costly, sub-optimal for the target cell, and also leads to toxicity and off-target side effects [16]. BMP-7 has been previously investigated as a treatment for OA in Phase I and Phase II clinical trials. The Phase I trial of BMP-7 showed a trend to a symptom response for knee OA with a lack of dose-limiting toxicity [48]. However, the results from the Phase II studies were not published and no further studies have been announced. Currently there are no BMP-2 products in clinical trials for the treatment of OA.

An injectable combination of BMP protein and PODS® crystals addresses many of these limitations of conventional growth factors by delivering therapeutic efficacy at lower doses of BMP over long periods of time. Therefore, PODS® technology has the potential to provide a convenient, off-the-shelf outpatient therapy for early OA, addressing a currently unmet medical need and reducing a significant healthcare burden. In conclusion, the use of the PODS® technology to provide sustained release of BMP-2 or BMP-7 at a reduced dose is a novel and clinically-relevant approach to develop a therapy for the treatment of cartilage defects associated with OA. Prior to translation, additional studies will be required to demonstrate efficacy in large animal models.

## Author contributions

CW and CM collected and assembled the data of *in-vitro* experiments. FH conducted the small animal study, collected and assembled *in vivo* data, CW, CP, AM, MJ, FH designed the study, analysed and interpretated the data and prepared the manuscript, All authors provided final approval of the article prior to submission.

## Funding information

This study was supported by an 10.13039/501100006041Innovate UK grant titled “constrained crystalline growth factors for therapy of synovial joint surface defects” (project #102848). The funding provider had no influence over the study design nor data analysis or interpretation.

## Declaration of competing interest

CW, CM, CP and MJ work for the company Cell Guidance Systems (CellGS) developing PODS® proteins. FH is a scientific advisor to CellGS and AM has no affiliation with CellGS or other commercial interests related to this study. The work reported in this study is the subject of a patent application.
